# Comprehensive evaluation of the response to aluminum stress in olive tree (*Olea europaea* L.)

**DOI:** 10.3389/fpls.2022.968499

**Published:** 2022-07-28

**Authors:** Erli Niu, Song Gao, Xiaomin Yu, Ali Soleimani, Shenlong Zhu

**Affiliations:** ^1^Key Laboratory of Digital Dry Land Crops of Zhejiang Province, Institute of Crops and Nuclear Technology Utilization, Zhejiang Academy of Agricultural Sciences, Hangzhou, China; ^2^Faculty of Agriculture, University of Zanjan, Zanjan, Iran

**Keywords:** olive (*Olea europaea* L.), aluminum tolerance, germplasm evaluation, metabolome, transcriptome

## Abstract

Olive (*Olea europaea* L.) is an ancient tree species in the Mediterranean, but the lack of knowledge about aluminum-resistant varieties limits its introduction to acidic soil. The objective of this study was to have a comprehensive evaluation of the response to aluminum stress in olive tree at germplasm, metabolome, and transcriptome levels. In this experiment, seedlings of 97 olive germplasm with 1.0–3.0 cm roots and two leaves were treated with 50 μM Al^3+^ (pH = 5.0). By factor analysis of the traits of defoliation rate, rooting rate, length of extended root, and length of new root, 97 germplasm were classified into five different groups according to their diverse responses to aluminum stress: 5 highly resistant (5.15%), 30 moderately resistant (30.93%), 31 general (31.96%), 23 moderately sensitive (23.71%), and 8 highly sensitive (8.25%) germplasm. The three most sensitive and three most resistant germplasm were further used for metabolome and transcriptome analysis. Exposed to aluminum stress, 96 differentially accumulated metabolites (DAMs)/4,845 differentially expressed genes (DEGs) and 66 DAMs/2,752 DEGs were identified in highly sensitive and resistant germplasm, respectively. Using multi-omics technology, the pathways and related DAMs/DEGs involved in cell wall/cytoplasm receptors, reactive oxygen species balance, hormone induction, synthesis of organic acids, Al^3+^ transport, and synthesis of metabolites were identified to mainly regulate the response to aluminum stress in olive. This study provides a theoretical guide and prior germplasm and genes for further genetic improvement of aluminum tolerance in the olive tree.

## Introduction

Acidic soil accounts for more than 40% of the arable land in the world, mainly in developing countries in tropical, subtropical, or temperate regions (Koehian, 1995). In addition to natural acidification, acid deposition, the application of acidic fertilizers, and the continuous cultivation of legumes can all aggravate the acidification of soil (Koehian, 1995; Poschenrieder et al., 2008; Liu et al., 2014). It has been estimated that aluminum toxicity is the major negative factor in acidic soil. When the soil pH is less than 5.0, aluminum mainly exists as soluble Al^3+^, which directly poisons plants, inhibits the growth of crops, and even reduces crop production (Poschenrieder et al., 2008; Liu et al., 2014). Breeders have tried a series of tactics to mitigate this. For example, lime can improve the soil pH and alleviate aluminum toxicity to a certain extent, but its use greatly increases costs and destroys the physiological characteristics of the soil. Therefore, in light of costs, production benefits, and ecological sustainability, understanding the response of plants to aluminum stress, evaluating of aluminum-resistant germplasm, and breeding new cultivars are the fundamental ways of solving the problem of plant intolerance to aluminum stress.

The root, the primary target of aluminum toxicity, is a key indicator to measure the tolerance of plants to aluminum stress (Poschenrieder et al., 2008). Aluminum toxicity has been widely studied in herbaceous plants, in particular rice (*Oryza sativa*), wheat (*Triticum aestivum*), sorghum (*Sorghum vulgare*), and barley (*Hordeum vulgare*). Rice is the most aluminum-tolerant species among small grain cereal crops (Li et al., 2017). Researchers screened different rice genotypes treated with 1 mM and 160 μM Al^3+^ for aluminum tolerance by determining total root growth, primary root growth, longest root growth, and root dry weight (Famoso et al., 2011; Tao et al., 2018). Longest root length and total root length were recorded to identify the tolerance of 166 barley accessions to 5 mM Al^3+^ (Cai et al., 2013). Tea (*Camellia sinensis*) is capable of withstanding aluminum stress at high concentrations without toxicity and in fact prefers acidic soils (pH 4.0–5.5) (Li et al., 2017; Ding et al., 2021). Mechanisms of resistance to aluminum stress in plants are categorized as exclusion (avoidance) and internal tolerance (Inostroza-Blancheteau et al., 2012; Liu et al., 2014; Pereira and Ryan, 2019). Exclusion prevents aluminum from entering the growing root through fixation of the cell wall; an increase in rhizosphere pH; or the secretion of organic acids (malic acid and citric acid), polyphenols, and phosphates. In contrast, internal tolerance allows the uptake of aluminum but can detoxify the plant through the compartmentalization of vacuoles or chelation of organic acids, proteins, and other organic compounds (Inostroza-Blancheteau et al., 2012; Liu et al., 2014; Pereira and Ryan, 2019). Although aluminum tolerance in plants is complex and not fully understood, more and more excellent genes have been identified with the development of multi-omics techniques. Two organic acid transporters, aluminum-activated malate transporter (ALMT) and multidrug and toxic compound extrusion (MATE), are critical resistance genes that can mediate malate and citrate effluxes for the secretion of organic acids (Sasaki et al., 2004; Magalhaes et al., 2007). Natural resistance-associated macrophage protein (Nramp) and vacuolar ATP-binding cassette (ABC) transporter are responsible for transporting aluminum ions and removing aluminum from the cell wall, and their mutants exhibit high sensitivity to aluminum (Xia et al., 2010; Huang et al., 2012). Aluminum toxicity can also trigger reactive oxygen species (ROS), Ca^2+^ channels, cell wall formation, and other metabolic processes, which require further exploration (Rengel and Zhang, 2003; Yamamoto et al., 2003; Liu et al., 2014; Ding et al., 2021).

Olive (*Olea europaea* L.), the second largest woody oil tree, has been cultivated in Mediterranean countries for about 6,000 years. Because of the redundant unsaturated fatty acids, polyphenols, and bioactive substances in olive oil, it has expanded and been introduced in more than 40 countries (Pérez-Jiménez et al., 2010; Rigacci and Stefani, 2016). Under the long-term domestication of Mediterranean environment, the olive tree is characterized by a resistance to barrenness, drought, and wind and a preference for neutral or slightly alkaline soil and sunlight (Kaniewski et al., 2012; Muzzalupo, 2012). However, the aluminum tolerance of olive germplasm and resistant cultivars has never been studied, which presents potential obstacles to the emerging introduction of the tree to areas with acidic soil. Moreover, available genome data from two cultivated (*O. europaea* cv. Farga and *O. europaea* cv. Arbequina) and one wild (*O. europaea* var. sylvestris) olive trees provide an effective way of identifying the molecular mechanisms involved in morphogenesis (Cruz et al., 2016; Unver et al., 2017; Rao et al., 2021). Researches on fruit development, fatty acid synthesis, and response to drought or salt or heat is plentiful (Rao et al., 2019; Tsamir-Rimon et al., 2020; Valente et al., 2020), but little is known about the response of the olive tree to aluminum stress.

In the present study, we investigated the comprehensive response of the olive tree to aluminum stress at germplasm, metabolome, and transcriptome levels. An effective method was firstly established to evaluate the aluminum tolerance of olive tree and classified 97 germplasm into different groups according to their diverse responses to aluminum stress. Subsequently, resistant and sensitive germplasm were used for further multi-omics (metabolome and transcriptome) analysis. It screened the resistant germplasm for potential use in breeding and clarified the important regulation mechanism of the responses to aluminum stress to contribute to the genetic improvement of the olive tree for aluminum tolerance.

## Materials and methods

### Plant materials and breeding

A total of 97 olive germplasm, including 94 cultivars (*O. europaea* subsp. *europaea*), 1 wild olive (oleaster), 1 *Cuspidata* subspecies, and 1 progeny of the cross of *Cuspidata* and cultivated olive were analyzed in this study. Of the germplasm, 44 were originally collected from Spain, 32 came from Italy, 8 from China, and the remaining 13 from Greece (*n* = 5), France (*n* = 4), Azerbaijan (*n* = 1), Portugal (*n* = 1), Tunisia (*n* = 1), and Algeria (*n* = 1) (Supplementary Table 1). All germplasm were grown in the experimental field of Zhejiang Academy of Agricultural Sciences in a 2.0 × 3.0 m space on average.

### Phenotyping for aluminum tolerance

All germplasm were propagated by cuttage in perlite. About 3 months later, when all cuttings had new roots of 1.0–3.0 cm and two leaves, experiments were performed to test their response to aluminum solution. All seedlings were cultured in 1/2 modified Hoagland solution (Ali et al., 2011) and treated with 50 μM AlCl_3_ (pH = 5.0). After 2 weeks, four parameters related to the response to aluminum stress in woody plant were measured: defoliation rate, rooting rate, length of extended root, and length of new root. The extended root and new root refer to the original root that continued to grow and to roots newly raised after treatment, respectively. Each germplasm contained 30 seedlings as biological replicates, and all trials were conducted under long-day conditions (16 h light/8 h dark cycle) at 26°C. Defoliation rate, length of extended root, and length of new root were calculated from the average of individual seedlings, and rooting rate was calculated as follows: rooting rate = (number of seedlings with rooting/30) × 100%.

### Factor analysis for the evaluation of aluminum tolerance

To evaluate the aluminum tolerance of the olive germplasm, factor analysis was conducted using SPSS software. First, Kaiser–Meyer–Olkin values and Bartlett’s statistic were calculated for the selected variables. Factors were subsequently extracted according to the cumulative contribution rate more than 85% rule. Finally, the comprehensive evaluation of aluminum tolerance was obtained based on the cluster analysis of factor scores with between-groups linkage method (Hou et al., 2018).

### Ultrahigh performance liquid chromatography-mass spectrometry analysis and determination of metabolites

Based on the results of the factor analysis, the three most resistant and three most sensitive germplasm were treated with 50 μM AlCl_3_ (pH = 5.0) again with 0 μM AlCl_3_ (pH = 7.0) as a control. After 2 weeks, root tips (∼1.0 cm) were sampled, frozen in liquid nitrogen, and stored in a freezer at –80°C for metabolome and transcriptome analysis. Samples of the three most resistant germplasm treated with 0 μM AlCl_3_ (pH = 7.0) and 50 μM AlCl_3_ (pH = 5.0) were mixed, respectively, and recoded as the R and RT groups. Samples of the three most sensitive germplasm treated with 0 μM AlCl_3_ (pH = 7.0) and 50 μM AlCl_3_ (pH = 5.0) were mixed, respectively, and recoded as the S and ST groups.

An ultrahigh performance liquid chromatography-mass spectrometry (UHPLC-MS) analysis was conducted with six independent biological replicates. Briefly, 50 mg freeze-dried and crushed samples were transferred to an Eppendorf tube and extracted with 500 μl methanol/water solution (v:v = 3:1). The mixture was homogenized at 35 Hz for 4 min and sonicated for 5 min in an ice-water bath three times. After extraction overnight at 4°C on a shaker, the samples were centrifuged at 12,000 rpm for 15 min at 4°C. The supernatants were filtered through a 0.22-μm microporous membrane and diluted 10 times with extract solution containing an internal standard. A 50-μl sample was stored at –80°C for the UHPLC-MS analysis.

A UHPLC-MS analysis was performed with an ExionLC system and SCIEX QTrap 6500 by Biotree Biotech Co., Ltd. (Shanghai, China). SCIEX analyst (v1.6.3) was used for MRM data acquisition and processing. MSconventer and an in-house R program were used for file conversion and annotation, respectively. Relative concentrations of metabolites were determined by peak area (mm^2^), and the mass spectra were then compared to known and commercially available mass spectral libraries. Principal component analysis and orthogonal projections to latent structures discriminant analysis were then performed with SIMCA16.0.2 (Sartorius Stedim Data Analytics, Umea, Sweden). Differentially accumulated metabolites (DAMs) with variable importance in the projection (VIP) > 1.0 and *p* < 0.05 (Student’s test) were fed into the Kyoto Encyclopedia of Genes and Genomes (KEGG)^1^ and MetaboAnalyst^2^ databases for pathways analysis.

### RNA-sequencing and enrichment of differentially expressed genes

Four root samples from the R, RT, S, and ST groups were examined with RNA-sequencing (RNA-seq). Total RNA was obtained from the root tips (∼1.0 cm) using CTAB with three biological replicates. Gel electrophoresis and a NanoDrop spectrophotometer (Thermo Fisher Scientific, Wilmington, DE, United States) were used to quantify RNA. Library DNA was checked for concentration and size distribution with an Agilent2100 Bioanalyzer and then sequenced with an Illumina NovaSeq 6000 System according to the manufacturer’s instructions. The raw data had been submitted as PRJNA818114 to the National Center for Biotechnology Information.^3^

To explore differentially expressed genes (DEGs), Python was used to map the reads to the olive reference genome *O. europaea* cv. Farga (Cruz et al., 2016), and the expression of each gene was represented as transcripts per kilobase of exon model per million mapped reads (TPM). DEGs were identified with Cuffdiff and were required to have a two-fold change and *Q*-value ≤ 0.01. Gene Ontology and KEGG pathway analyses were conducted with agirGO and KOBAS (Du et al., 2010). In addition, 20 genes were randomly selected for quantitative real-time PCR (qRT-PCR) to analyze of the Pearson correlation between RNA-seq and qRT-PCR. The *OeActin* (OE6A099235) gene was used as the endogenous control and the primers of 20 genes are listed in Supplementary Table 2 (Niu et al., 2022).

### Correlation analysis of metabolome and transcriptome data

Upregulated DEGs and DAMs in RT vs. R group were used to conduct correlation analyses between metabolome and transcriptome data. The Spearman method and Cytoscape (v3.3.0) were used to analyze correlation coefficients and correlation networks with | correlation coefficient | ≥ 60.0%. Clipart downloaded from PinClipart^4^ was arranged to draw a diagram of the olive tree.

## Results

### Phenotypic variation in traits of olive germplasm related to aluminum tolerance

To screen out excellent olive germplasm resistance to aluminum toxicity in acidic soil, 97 olive germplasm collected from nine countries were propagated by cutting. Seedlings with 1.0–3.0 cm roots and two leaves of different germplasm were treated with 50 μM Al^3+^ (pH = 5.0). After 2 weeks, four indicators related to aluminum tolerance traits including defoliation rate, rooting rate, length of extended root, and length of new root were used to evaluate the responses of the seedlings to aluminum stress (Table 1).

**TABLE 1:** Various traits related to aluminum tolerance.

Traits	Minimum value	Maximum value	Mean value	SD	CV
Defoliation rate/%	0	48.33	14.29	6.83	57.63
Rooting rate/%	33.33	100	92.05	–	–
Length of extended root/cm	2.52	16.35	7.18	5.07	0.72
Length of new root/cm	0.19	11.74	4.50	4.64	1.14

Traits of 97 olive germplasm after treatment with 50 μM AlCl_3_ (pH = 5.0) were measured. Each germplasm contained 30 seedlings as biological replicates (n = 30). SD, standard deviation; CV, coefficient of variation.

The average defoliation rate and rooting rate were 14.29 and 92.05%, ranging from 0 to 48.33% and from 33.33 to 100%, respectively (Table 1). The average length of extended root was 7.18 cm (2.52–16.35 cm), and the average length of new root was 4.50 cm (0.19–11.74 cm). This means that compared to the new root, the extended root of olive germplasm grew much faster with a lower coefficient of variation in aluminum solution (Table 1). Correlation analysis revealed that length of extended root and length of new root were positively correlated (Pearson index = 0.506, *p* < 0.001), although no other traits were correlated (| Pearson index| ≥ 0.5).

### Comprehensive evaluation of aluminum tolerance

**FIGURE 1:**
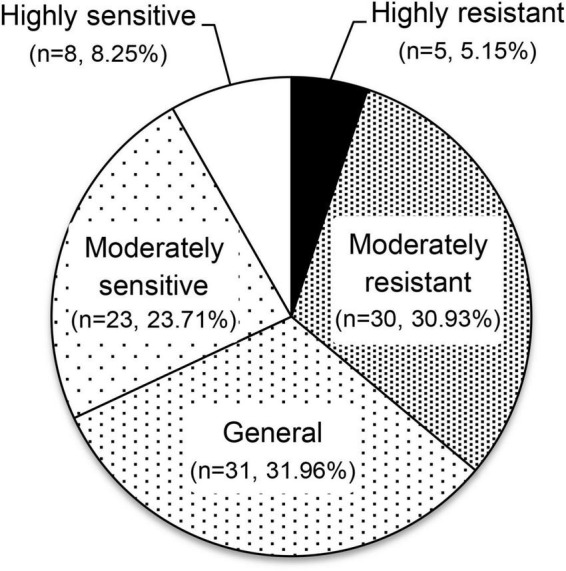
Comprehensive evaluation of aluminum tolerance in 97 olive germplasm. The 97 olive germplasm were classified into five different groups according to their diverse responses to aluminum stress (50 μM AlCl_3_, pH = 5.0) by factor analysis with 30 biological replicates included.

To evaluate the aluminum tolerance of the 97 olive germplasm, factor analysis of traits related to aluminum tolerance was conducted. The Kaiser–Meyer–Olkin value was 0.712 (> 0.5), and Bartlett’s statistic was < 0.05, which indicated that the factor analysis could be used for further study. A total of three independent factors accounting for 88.52% of the total variance were generated. Factor 1 represented the two indices of root length: length of extended root, and length of new root. Factors 2 and 3 represented the vitality of the leaves and roots, including defoliation rate and rooting rate, respectively. Finally, comprehensive *F*-values for each individual germplasm were calculated, based on which the 97 olive germplasm were clustered into five independent groups (Figure 1 and Supplementary Table 1). Five germplasm (5.15%) were in the highly resistant group, with F factors values of 0.97–1.29: Arroniz, Biancolilla, Blanqueta, I-79, and Royeta de Asque. A total of 30 (30.93%), 31 (31.96%), and 23 (23.71%) germplasm fell into the moderately resistant, general, and moderately sensitive groups, with *F*-values of 0.26 to 0.83, –0.19 to 0.21, and –0.73 to –0.25, respectively. Eight germplasm (8.25%) were in the highly sensitive group, with *F*-values of –1.70 to –0.84: Koroneiki, Manzanilla, Maurino, Moraiolo, Nociara, Pendolino, Piga, and Zen.

### Ultrahigh performance liquid chromatography-mass spectrometry analysis and exploration of metabolites

To investigate differences in metabolites and transcription, the three most resistant and three most sensitive germplasm were treated with 50 μM Al^3+^ (pH = 5.0) again and recoded as the R and S groups. As a control, these germplasm were also treated with 0 μM Al^3+^ (pH = 7.0) and recoded as the RT and ST groups. After 2 weeks, equal amounts of root tip (∼1.0 cm) from the R, S, RT, and ST groups were sampled to conduct untargeted metabolome analysis using an UHPLC-MS approach. Ultimately, a total of 604 metabolites were identified in each group, including carbohydrates, lipids/fatty acyls, flavonoids, phytohormones, organic acids, and derivatives, and so on.

**FIGURE 2:**
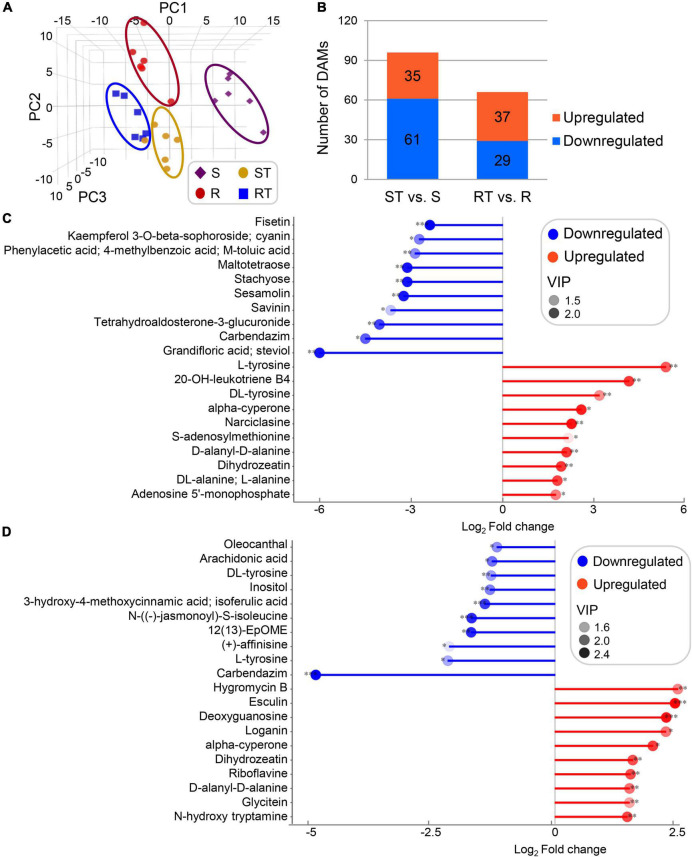
Global statistics on differentially accumulated metabolites (DAMs) derived from germplasm highly sensitive and highly resistant to aluminum. **(A,B)** Principal component analysis (PCA) score plot and total numbers of DAMs. **(C,D)** DAMs with the top 10 fold change of ST vs. S group **(C)** and RT vs. R group **(D)**. DAMs were identified according to the cutoffs variable importance in the projection (VIP) > 1 and *p* < 0.05. S, highly sensitive germplasm; R, highly resistant germplasm; ST, highly sensitive germplasm after treatment; RT, highly resistant germplasm after treatment.

Principal component analysis revealed the obvious differences between groups (Figure 2A). Using the cutoffs VIP > 1 and *p* < 0.05, 35/61 (upregulated/downregulated) and 37/29 DAMs were obtained in ST vs. S group and RT vs. R group, respectively (Figure 2B). In ST vs. S group, the top three upregulated/downregulated DAMs in terms of fold change were L-tyrosine, 20-OH-leukotriene B4, DL-tyrosine/grandifloric acid/steviol, carbendazim, and tetrahydroaldosterone-3-glucuronide, involved in the biosynthesis of amino acids, fatty acyls, alkaloids/diterpenoids, benzimidazoles, and organooxygen compounds (Figure 2C and Supplementary Table 3). In RT vs. R group, the top three upregulated/downregulated DAMs in terms of fold change were hygromycin B, esculin, deoxyguanosine/carbendazim, L-tyrosine, and (+)-affinisine, involved in the biosynthesis of organooxygen compounds, coumarins, purine/benzimidazoles, amino acids, and alkaloids (Figure 2D and Supplementary Table 3). Of them, L-tyrosine was mapped to various pathways, including aminoacyl-tRNA biosynthesis; phenylalanine, tyrosine, and tryptophan biosynthesis; isoquinoline alkaloid biosynthesis; tyrosine metabolism; and ubiquinone and other terpenoid-quinone biosynthesis.

Comprehensive analysis of the DAMs and pathways in ST vs. S group and RT vs. R group showed that the DAMs could be clustered into four types (Table 2 and Supplementary Table 3). Type I included 10 DAMs that were commonly upregulated after aluminum treatment in both the R and S groups: 4-pyridoxolactone, adenosine 3′-monophosphate, alpha-cyperone, D-alanyl-D-alanine, dihydrozeatin, esculin, lecanoric acid, narciclasine, nicotinamide, and tabernanthine. Type II included 10 DAMs that were commonly downregulated after aluminum treatment in both the R and S groups: 12(13)-EpOME, 3-hydroxy-4-methoxycinnamic acid/isoferulic acid, 4-methylumbelliferone, 5-methyl-2-furaldehyde, arachidonic acid, carbendazim, ethyl gallate, L-carnitine, N-[(–)-jasmonoyl]-S-isoleucine, and oleocanthal. Type III included 76 DAMs that were upregulated in RT vs. R group but downregulated (*n* = 2) or not significantly different (*n* = 25) in ST vs. S group, and not significantly different in RT vs. R group but downregulated in ST vs. S group (*n* = 49). Of these, the two DAMs with opposite accumulation trends in RT vs. R group and ST vs. S group were alexine/australine and serotonin, belonging to alkaloid and tryptamine derivative, respectively. Type IV included 39 DAMs that were downregulated in RT vs. R group but upregulated (*n* = 5) or not significantly different (*n* = 14) in ST vs. S group, and not significantly different in RT vs. R group but upregulated in ST vs. S group (*n* = 20). The five DAMs (3-methy-L-histidine, DL-tyrosine, L-histidine, L-tyrosine, and tryptophan/L-tryptophan/D-tryptophan) with opposite accumulation trends were amino acids or alkaloid derivatives (Table 2 and Supplementary Table 3). Pathways analysis of the DAMs showed that in both the R and S groups, 15 pathways were enriched after aluminum treatment: aminoacyl-tRNA biosynthesis; arginine and proline metabolism; biosynthesis of unsaturated fatty acids; glucosinolate biosynthesis; glycine, serine, and threonine metabolism; histidine metabolism; indole alkaloid biosynthesis; isoquinoline alkaloid biosynthesis; nicotinate and nicotinamide metabolism; phenylalanine, tyrosine, and tryptophan biosynthesis; purine metabolism; tryptophan metabolism; tyrosine metabolism; ubiquinone and other terpenoid-quinone biosynthesis; and zeatin biosynthesis. Six pathways (alanine, aspartate, and glutamate metabolism; carotenoid biosynthesis; glutathione metabolism; riboflavin metabolism; steroid biosynthesis; and valine, leucine, and isoleucine biosynthesis) were enriched in RT vs. R group, but not in ST vs. S group, and 15 pathways were only enriched in ST vs. S group but not in RT vs. R group (Supplementary Table 3).

**TABLE 2:** Comprehensive analysis of differentially accumulated metabolites (DAMs) and differentially expressed genes (DEGs) in RT vs. R group and ST vs. S group.

Types	RT vs. R	ST vs. S	Number of DAMs	Number of DEGs
Type I	Up	Up	10	78
Type II	Down	Down	10	35
Type III	Up	Down	2	33
	Up	–	25	896
	–	Down	49	1,457
Type IV	Down	Up	5	639
	Down	–	14	1,071
	–	Up	20	2,603

DAMs were fed by ultrahigh performance liquid chromatography-mass spectrometry analysis (n = 6) with the cutoffs variable importance in the projection (VIP) > 1 and p < 0.05, and DEGs were obtained by RNA-sequencing (n = 3) with the cutoffs Q-value ≤ 0.01 and log_2_| fold change| ≥ 1. R, highly resistant germplasm; S, highly sensitive germplasm; RT, highly resistant germplasm after treatment; ST, highly sensitive germplasm after treatment.

### Transcriptome analysis and identification of elite alleles

**FIGURE 3:**
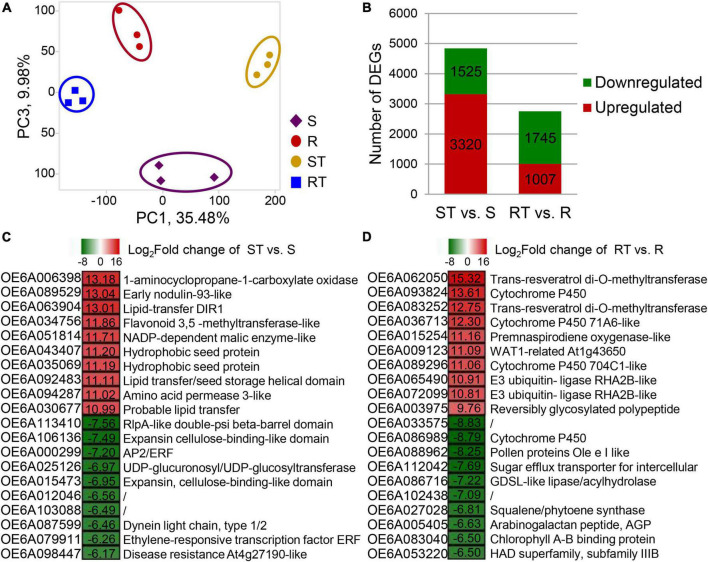
Global statistics on differentially expressed genes (DEGs) identified from germplasm highly sensitive and highly resistant to aluminum. **(A,B)** Principal component analysis (PCA) score plot and total numbers of DEGs. **(C,D)** DEGs with the top 10 fold change in ST vs. S group **(C)** and RT vs. R group **(D)**. The expression of each gene was calculated as transcripts per kilobase of exon model per million mapped reads (TPM). DEGs were identified with Cuffdiff and were required to have a twofold change with false discovery rate (FDR) < 0.05. S, highly sensitive germplasm; R, highly resistant germplasm; ST, highly sensitive germplasm after treatment; RT, highly resistant germplasm after treatment.

RNA-seq analysis of the S, R, ST, and RT groups was performed to identify candidate genes related to the response to aluminum stress in olive tree. A total of 42.64 million clean reads were obtained and aligned with the olive reference sequences from *O. europaea* cv. Farga (Cruz et al., 2016) with a mapping rate of 91.12% (Supplementary Table 4). TPM was used to calculate the expression of each gene, and had a Pearson correlation coefficient of 0.91 with the results from qRT-PCR (Supplementary Figure 1). Using the cutoffs *Q*-value ≤ 0.01 and log_2_| fold change| ≥ 1, 3,320/1,525, and 1,007/1,745 upregulated/downregulated DEGs were identified in ST vs. S group and RT vs. R group, respectively (Figures 3A,B). Overall, the total number of DEGs in RT vs. R group were less than the number in ST vs. S group. In ST vs. S group, the top three upregulated genes were coding 1-aminocyclopropane-1-carboxylate oxidase (OE6A006398), early nodulin-93-like (OE6A089529), and lipid-transfer DIR1 (OE6A063904), whereas the top three downregulated genes were involved in RlpA-like double-psi beta-barrel domain (OE6A113410), expansin cellulose-binding-like domain (OE6A106136), and AP2/ERF (OE6A000299) (Figure 3C). In RT vs. R group, the top three upregulated genes were OE6A062050 (trans-resveratrol di-O-methyltransferases), OE6A093824 (cytochrome P450), and OE6A083252 (trans-resveratrol di-O-methyltransferases), whereas the top three downregulated genes were OE6A033575 (unknown function), OE6A086989 (cytochrome P450), and OE6A088962 (pollen proteins Ole e I like) (Figure 3D).

In RT vs. R group and ST vs. S group, 78 and 35 DEGs were commonly upregulated and downregulated, respectively, after aluminum treatment (Table 2). The 78 upregulated DEGs were mainly involved in terpene synthesis (vetispiradiene synthesis), oxidoreductase activity (multicopper oxidase, premnaspirodiene oxygenase, cytochrome P450, β-amyrin 28-oxidase, and 1-aminocyclopropane-1-carboxylate oxidase homolog 1), protein kinase activity (receptor kinase, LRR), and organic acid metabolism (oxalate ligase). Moreover, 929 DEGs were only upregulated in RT vs. R group, of which 33 were downregulated in ST vs. S group and 896 were not significantly different in ST vs. S group. The 33 DEGs included multiple transcription factors (ERF, zinc finger, and NAC), protein kinases, and transporters. For example, the three genes OE6A073725, OE6A008137, and OE6A009890 encoding receptor kinase, detoxification 49, and glutathione gamma-glutamylcysteinyltransferase 1, respectively, were upregulated 53. 2-, 22. 3-, and 16.1-fold in RT vs. R group but downregulated 0. 47-, 0. 22-, and 0.34-fold in ST vs. S group (Table 2). A total of 1710 DEGs were only downregulated in RT vs. R group, of which 639 were upregulated in ST vs. S group and 1,071 were not significantly different in ST vs. S group. Moreover, 4,060 DEGs were unchanged in RT vs. R group but downregulated or upregulated in ST vs. S group (Table 2).

### Integrative analysis of metabolome and transcriptome data

**FIGURE 4:**
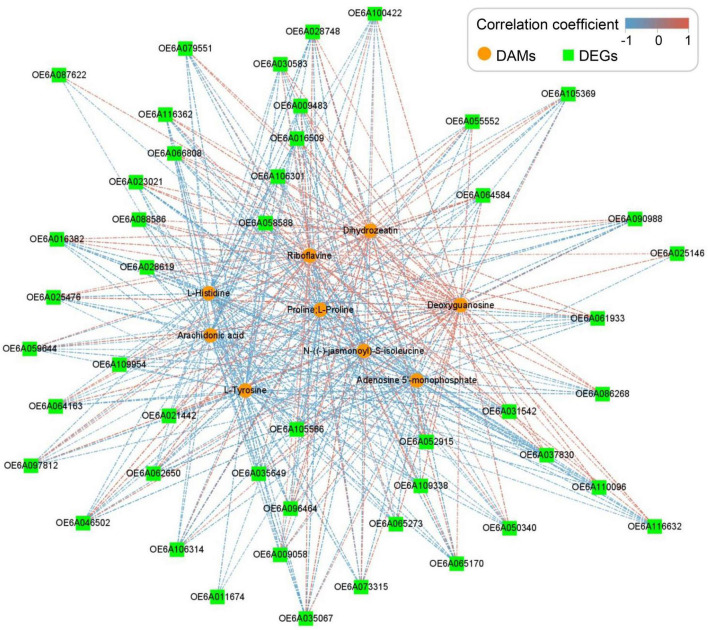
Network analysis of correlations between differentially accumulated metabolites (DAMs) and differentially expressed genes (DEGs). The upregulated genes and metabolites identified from the highly resistant germplasm after treatment (RT) vs. highly resistant germplasm (R) group were used for correlation analysis in Cytoscape v3.3.0 with | correlation coefficient| ≥ 60.0%. Nodes represent DAMs and DEGs, and lines represent the correlations between them.

Correlation analysis of metabolome and transcriptome data of RT vs. R group was performed to identify upregulated genes and metabolites. Seven pathways showed strong correlations (> 50 hits) between each DEG and DAM (Supplementary Table 5). The top three hit pathways were oeu04075 (plant hormone signal transduction), oeu00940 (phenylpropanoid biosynthesis), and oeu04141 (protein processing in endoplasmic reticulum). Phenylpropanoid biosynthesis includes the biosynthesis of flavonoids and lignins, which has been proven to be involved in plant defense (Polturak and Osbourn, 2021). Further analysis showed that carbon metabolism, oxidative phosphorylation, citrate cycle, fatty acid synthesis, mitogen-activated protein kinase (MAPK)-signaling pathway, and ABC transporters were also identified in both metabolome and transcriptome comparison (Figure 4).

## Discussion

### Aluminum tolerance of different olive germplasm resources

Olive is an ancient evergreen fruit tree with about 6,000 years of cultivation history in the Mediterranean region. It has generally adapted well to neutral to weakly alkaline soil. In acidic soil, aluminum toxicity can cause problems such as blocked vegetative growth, serious defoliation, and even premature death (Pérez-Jiménez et al., 2010; Kaniewski et al., 2012; Muzzalupo, 2012). Unfortunately, no related germplasm or mechanisms have been elucidated until now. In this study, we were the first to establish an effective method of evaluating the aluminum tolerance of olive germplasm by culturing young seedlings in Hoagland solution. This method included criteria for seedling selection, treatment designs and index parameters, and consideration of other factors such as pH and root growth characteristics. The results showed that it is a simple, efficient, and accurate method that enables large-scale assessment of olive germplasm independent of environmental influences and acts as a useful technical reference for further research on aluminum tolerance.

There were clearly different levels of tolerance to aluminum stress among the 97 olive germplasm tested. Cluster analysis showed that germplasm could be classified into five different groups according to their diverse responses to aluminum stress: highly resistant (*n* = 5, 5.15%), moderately resistant (*n* = 30, 30.93%), general (*n* = 31, 31.96%), moderately sensitive (*n* = 23, 23.71%) and highly sensitive (*n* = 8, 8.25%), indicating wide genetic diversity. Resistant germplasm could be used directly in field trials for cutting or grafting, or to improve the aluminum tolerance of olive cultivars through cross-breeding. It is worth noting that the results seemed to reflect an integrated treatment of aluminum, acid, and waterlogging stress because of the hydroculture method used. For example, Arbequina has been widely introduced in acidic soil and presents a good yield (Rao et al., 2021). Yet after waterlogging treatment, it showed poor shoot growth and a low survival rate (< 90.0%). In addition, three germplasm Olea Cuspidata Wall, Sylvestris, and Zhonglan, belonging to *Cuspidata* subspecies, wild olive, and the progeny of the cross of *Cuspidata* and cultivated olive, respectively, showed obvious differences from cultivated olive in both morphology and genetics, so their aluminum tolerance should be evaluated alone according to individual developmental traits.

### Genetic response to aluminum stress of olive tree

**FIGURE 5:**
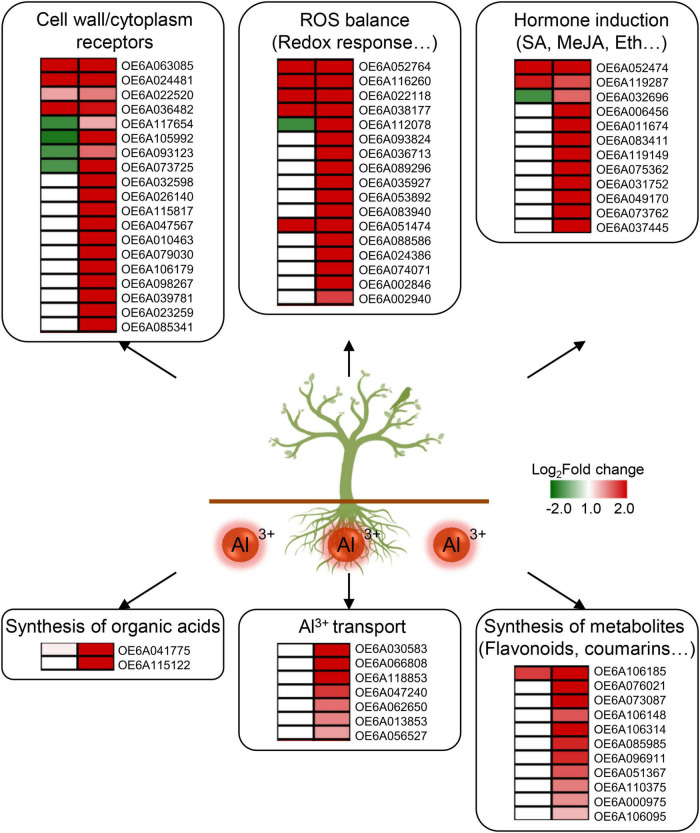
Key pathways and differentially expressed genes (DEGs) in the response to aluminum stress in olive tree. Six pathways and related DEGs including the cell wall/cytoplasm receptors, ROS balance, hormone induction, synthesis of organic acids, Al^3+^ transport, and synthesis of metabolites, are mainly involved in the response to aluminum stress in olive. Expression changes on the left side and right side represent ST vs. S group and RT vs. R group, respectively. S, highly sensitive germplasm; R, highly resistant germplasm; ST, highly sensitive germplasm after treatment; RT, highly resistant germplasm after treatment; ROS, reactive oxygen species; SA, salicylic acid; MeJA, methyl jasmonate; Eth, ethylene.

Integrated transcriptome and metabolome analyses of the R and S groups were conducted to reveal key response pathways. Among the 96 DAMs and 4,845 DEGs in ST vs. S group and the 66 DAMs and 2,752 DEGs in RT vs. R group, receptor kinase sensor, redox response, hormone induction, and synthesis of organic acids, flavonoids, and coumarins mainly participated in the response to aluminum stress in the olive tree (Figure 5). Receptor kinase is considered a key regulator of signal transduction. It can receive extracellular signals and activate downstream proteins through phosphorylation or dephosphorylation. Meanwhile, stress also disturbs the ROS balance and further triggers a series of redox responses. In this study, various related DEGs were enriched under aluminum stress (Figure 5). Four transcripts of OE6A117654, OE6A105992, OE6A093123, and OE6A073725 encoding receptor kinases were upregulated to 15.8-fold in RT vs. R group but downregulated 0.4-fold in ST vs. S group. In the pathways related to redox response, genes involved in NADPH activity (OE6A052764 and OE6A116260) and cytochrome P450 (OE6A022118, OE6A038177, OE6A112078, etc.) were upregulated after aluminum treatment. Previous studies established that after treatment with ethylene, the roots of *Arabidopsis thaliana* became tolerant to low pH, which is involved in peroxidases-dependent cell wall modification (Graças et al., 2021). Here, several peroxidases were also upregulated after aluminum treatment in the R group, including OE6A051474, OE6A088586, OE6A024386, OE6A074071, OE6A002846, and OE6A002940 (Figure 5).

Salicylic acid, methyl jasmonate, and ethylene play inportant roles in the regulation of defense responses to diverse stresses, such as water, temperature, and salt (Iqbal et al., 2017; Mahmoud et al., 2021; Zhao et al., 2021). The accumulation of salicylic acid or methyl jasmonate can increase aluminum resistance in *Glycine max* and *Vaccinium corymbosum* (Liu, 2011; Ulloa-Inostroza et al., 2017). After aluminum treatment, the content of methyl jasmonate was significantly increased 1.68-fold in the R group, and the expression of OE6A052474 (a salicylic acid-binding protein) was upregulated in both the R and S groups (Figure 5 and Supplementary Table 3). Meanwhile, the ethylene-responsive transcription factors OE6A119287 and OE6A032696 were also upregulated in RT vs. R group (Figure 5). Moreover, content of N6-isopentenyladenosine, a kind of cytokinin, and expression of OE6A037445, an auxin-responsive SAUR68, were also upregulated in the R group after aluminum treatment (Figure 5 and Supplementary Table 3).

The secretion of organic acids can alleviate the toxicity of Al^3+^. Oxalate and malate have been proven to be responsible for both extra- and intracellular chelation of Al^3+^ (Inostroza-Blancheteau et al., 2012; Liu et al., 2014; Pereira and Ryan, 2019). In the present study, OE6A041775, an oxalate ligase, was upregulated in both RT vs. R and ST vs. S group. However, OE6A115122, a malate dehydrogenase, was only upregulated 8.79-fold in RT vs. R group, with no significant change in ST vs. S group. Similarly, seven ABC transporters (OE6A030583, OE6A066808, OE6A118853, OE6A047240, OE6A062650, OE6A013853, and OE6A056527) were upregulated 5.46-fold evenly by aluminum stress in the R group (Figure 5). This proves that these transcripts are important for enhancing secretion of organic acid in the olive tree.

Secondary metabolites are key signals to regulate plant growth, and their accumulation is significantly increased under multiple biological or abiotic stresses. Among them, flavonoids and coumarins play important roles in antioxidant activity and free radical scavenging, which can significantly enhance plant resistance to various stresses (Panat et al., 2016; Singh et al., 2020). Previous studies have found that scavenging rates of different flavonoids on hydroxyl radicals increased with their content in the order quercetin > baicalein > baicalin in *Scutellaria baicalensis* (Gao et al., 1999). Flavonoids, especially polyphenols, are good protectors of root elongation against Al^3+^ (Barceló and Poschenrieder, 2002). After aluminum treatment, contents of two flavonoids, deguelin and glycitein, increased 2.47- and 2.86-fold, respectively, in R group roots (Supplementary Table 3). Related genes involved in flavonoid biosynthetic processes were also upregulated, such as OE6A106185 (a 7-deoxyloganetin glucosyltransferase related to quercetin metabolism), OE6A076021 (an anthocyanidin 3-O-glucosyltransferase), OE6A073087 (a flavonoid 3-monooxygenase), OE6A106148 (a 2-hydroxyisoflavanone dehydratase), and seven UDP-glycosyltransferases (OE6A106314, OE6A085985, OE6A096911, OE6A051367, OE6A110375, OE6A000975, and OE6A106095) (Figure 5). This illustrates the essential role of flavonoids in resistance to aluminum stress in the olive tree. Coumarin is a plant antitoxin and also a precursor to lignin synthesis. In response to salinity or iron deficiency, coumarin can induce antioxidant defense, the glyoxalase system, and ion homeostasis (Perkowska et al., 2021). Here, the contents of three coumarins, 3,4-dihydrocoumarin, esculin and psoralidin increased 1. 88- and 5. 45-, and 1.70-fold, respectively, after aluminum treatment in the R group, but were not significantly changed in the S group (Supplementary Table 3).

All these pathways, including metabolites and transcripts, work together to protect the olive tree from aluminum stress. The response to aluminum stress is multi-dimensional, and more research effort is needed at the gene, protein, and metabolite levels (Pires and Dolan, 2010; Feng et al., 2020). Moreover, multiple transcription factors, such as ERF, bHLH, and WRKY, play essential roles in plant stress and also the response to the aluminum stress in the olive tree. The functional identification of candidate genes needs further attention, which could provide a reference for genetic improvement of aluminum resistance in the olive tree.

## Conclusion

In this study, an effective method of evaluating the aluminum tolerance of olive germplasm was established, which enabled large-scale identification in the seeding stage of the olive tree. Using factor analysis, 97 olive germplasm were classified into five different groups according to their diverse responses to aluminum stress: highly resistant (*n* = 5, 5.15%), moderately resistant (*n* = 30, 30.93%), general (*n* = 31, 31.96%) and moderately sensitive (*n* = 23, 23.71%), and highly sensitive (*n* = 8, 8.25%) germplasm. The three most sensitive and three most resistant germplasm were used for transcriptome and metabolome analysis. After treatment with 50 μM AlCl_3_ (pH = 5.0), 96 DAMs/4,845 DEGs and 66 DAMs/2,752 DEGs were identified in highly sensitive and resistant germplasm, respectively. Using multi-omics technology, six pathways and the related DAMs/DEGs seemed to be mainly involved in the response to aluminum stress in olive tree including the cell wall/cytoplasm receptors, ROS balance, hormone induction, synthesis of organic acids, Al^3+^ transport, and synthesis of metabolites. These results provide a theoretical guide and prior germplasm and genes for further genetic improvement of aluminum tolerance in olive tree.

## Data availability statement

The datasets presented in this study can be found in online repositories. The names of the repository/repositories and accession number(s) can be found in the article/Supplementary material.

## Author contributions

SZ and EN conceived and designed the study. EN, SG, and XY carried out the experiment and analyzed the data. EN wrote the manuscript. AS and SZ reviewed and revised the manuscript. All authors read and approved the final manuscript.

## Footnotes

1
http://www.genome.jp/kegg/

2
http://www.metaboanalyst.ca/

3
https://www.ncbi.nlm.nih.gov/

4
https://www.pinclipart.com/
