# Supplementary material for: Comprehensive evaluation of the response to aluminum stress in olive tree (Olea europaea L.)
Source: Front Plant Sci. 2022 Jul 28;13:968499. doi: 10.3389/fpls.2022.968499 (PMC9366337; doi:10.3389/fpls.2022.968499)
Supplement: Supplementary file 1 [file Data_Sheet_1.docx]

Supplementary Material


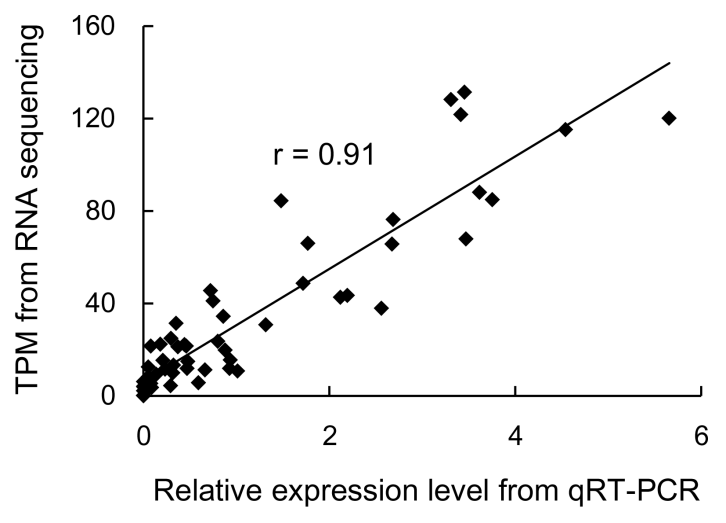


**Supplementary Figure 1.** Pearson correlation analysis of expression patterns between RNA-sequencing (RNA-seq) and quantitative real-time PCR (qRT-PCR). A total of 20 genes were randomly selected for Pearson correlation analysis. The x and y axes indicate the transcripts per kilobase of exon model per million mapped reads (TPM) calculated from RNA-seq data and relative expression level in qRT-PCR, respectively. The genes and corresponding primers are listed in Supplementary Table 2.

**Supplementary Table 1** Information on the 97 olive germplasm used in this study.

| Germplasm | Introduced country | Aluminum - tolerance^a^ | Germplasm | Introduced country | Aluminum - tolerance^a^ |
| --- | --- | --- | --- | --- | --- |
| Albareta | Spain | Moderately sensitive | Leccino | Italy | Moderately resistant |
| Alia | Spain | Moderately sensitive | Limona | Italy | Moderately sensitive |
| Alquezrana | Spain | General | Loaime | Spain | Moderately sensitive |
| Arbequina | Spain | General | Manzanilla | Italy | Highly sensitive |
| Arbosana | Spain | General | Manzanilla Greece | Spain | General |
| Arroniz | Spain | Highly resistant | Manzanilla sevillana | Spain | Moderately sensitive |
| Ascolana tenera | Italy | Moderately resistant | Maurino | Italy | Highly sensitive |
| Becarut | Spain | Moderately sensitive | Mochuto | Spain | Moderately resistant |
| Benizar | Spain | General | Moraiolo | Italy | Highly sensitive |
| Berat | Albania | Moderately resistant | Morcona | Italy | Moderately sensitive |
| Bianchera | Italy | General | N-79 | China | General |
| Biancolilla | Greece | Highly resistant | Nación | Spain | Moderately resistant |
| Blancal | France | Moderately sensitive | Negral de Bierge | Spain | General |
| Blanqueta | Spain | Highly resistant | Neral | Spain | General |
| Blanqueta | Spain | Moderately sensitive | Nevadillo fino | Spain | Moderately resistant |
| Bouteillan | France | General | Nevadillo negro | Spain | Moderately sensitive |
| Canino | Italy | Moderately sensitive | Nikitskii I | Azerbaijan | General |
| Carrasqueno | Spain | Moderately sensitive | Nocellara del belice | Italy | Moderately resistant |
| Castañero | Spain | Moderately sensitive | Nociara | Italy | Highly sensitive |
| Castellana | Spain | Moderately resistant | Nostrale di rigali | Italy | General |
| Cerruda | Spain | General | Olea Cuspidata Wall | China | Moderately sensitive |
| Chemlal de Kabylie | Algeria | Moderately resistant | Olivo de Caniles | Spain | Moderately resistant |
| Chenggu 32 | China | Moderately resistant | Olivon de Roda | Spain | Moderately resistant |
| Chétoui | Tunisia | Moderately resistant | Pendolino | Italy | Highly sensitive |
| Cipressino | Italy | General | Peranzana | Italy | Moderately sensitive |
| Cobrancosa | Portugal | General | Picholine | France | General |
| Cornequelo de jaén | Spain | Moderately resistant | Pico limón | Spain | General |
| Cornezuelo de Jaén | Spain | General | Picual | Spain | Moderately resistant |
| Cornicabra | Spain | General | Picudo de Labata | Spain | Moderately sensitive |
| Cortina | Italy | Moderately resistant | Piga | Spain | Highly sensitive |
| Cuquillo | Spain | Moderately sensitive | Redondilla | Spain | Moderately sensitive |
| Dolce agogia | Italy | General | Rogani | Greece | Moderately resistant |
| Dolce agogia selection | Italy | Moderately resistant | Rosciola | Italy | Moderately resistant |
| Ezhi 8 | China | Moderately resistant | Royeta de Asque | Spain | Highly resistant |
| Favolosa | Italy | Moderately resistant | Santa caterina | Italy | General |
| Fecciaro | Italy | Moderately sensitive | Sevillana de Labata | Spain | General |
| Frantoio | Italy | General | Skititta | Spain | Moderately resistant |
| Frantoio selezione | Italy | General | Sylvestris | Spain | General |
| Gentile di Chieti | Italy | General | Taggiasca | Italy | Moderately resistant |
| Grignan | Italy | General | Total de Cazorla | Spain | General |
| Grossa di Spagna | Italy | Moderately resistant | Verdeña | Spain | Moderately resistant |
| Hojiblanca | Spain | Moderately resistant | Verdial de badajoz | Spain | Moderately sensitive |
| Huaou 5 | China | Moderately resistant | Verdiell | Spain | Moderately sensitive |
| Huyette | France | General | Yuchan | Italy | General |
| I-77 | Italy | Moderately resistant | YT selection | China | General |
| I-79 | Italy | Highly resistant | Yuntai | China | Moderately sensitive |
| Konservolia | Greece | Moderately resistant | Zen | Italy | Highly sensitive |
| Koroneiki | Greece | Highly sensitive | Zhonglan | China | Moderately resistant |
| Largueta | Spain | Moderately sensitive |  |  |  |

*^a^ The diverse responses to aluminum stress were evaluated comprehensively using the factor analysis proposal and based on the cluster analysis of factor scores with between-groups linkage method (Hou et al., 2018).*

**Supplementary Table 2** Genes and corresponding primers for the quantitative real-time PCR analysis.

| Gene ID | Forward primer sequence (5'-3') | Reverse primer sequence (5'-3') |
| --- | --- | --- |
| OE6A117728 | GATCTGTATGGTAACATTGTCC | GGACCGGACTCATCATATTCG |
| OE6A020079 | CGAGGAGGATGGTGAATGGGAGG | GCCAATGGCTGCGATGGGCTC |
| OE6A096840 | GCTGGTGCCCTGATTGTGTGAGAG | GCTTCGTGATCTTCCAGACGAC |
| OE6A096894 | CGAACATATCGGTTCATAGTATTC | ATCCTGCTCTTTGGGACATTC |
| OE6A097054 | GATGAGCAAGAGAATGATAGGGC | CTTGATTTGGTTTCGAACACCATC |
| OE6A097112 | GCGTCACCATTGTTGGTGCTGTTTC | CTGATATCAATAAAATCTGGATC |
| OE6A097129 | GGTAGTGCACATGCTTCTCTC | CAAGTGACGTTCTGCTGCTCTGG |
| OE6A097144 | GTATGGTAAAACACGTGCCTC | CTACGAGCTTAGCGGTGAGTC |
| OE6A097254 | GAGATCACGCACTAGGGATTC | CTTATGATTCTGTTGCCTACCTGC |
| OE6A097308 | GTGGTGGCAGTTCTTGCAATCTTTC | GTTCCATGCTCTAGGTTTCTGC |
| OE6A097460 | GAGCCCGGCATTCTGCCCGTATC | GTAAAGACCCAAAGCTGCATG |
| OE6A069627 | TCTGATGTTTGTGTTATCGCCAC | AATCTCGATCCACAGTCGCC |
| OE6A051290 | TTGGTTAAGGGGAGCGTTGG | TGTCGCCTCCATTGCATGAT |
| OE6A116067 | AAGTTGGAAAGTGGACCCCA | GGTTCTCCAATTTGCCTAAAACC |
| OE6A118450 | GATGGAGTCCGAGATGAGACG | ACCATGCCTATTCTCTTCAGCT |
| OE6A020845 | ACACCCTAGATGGAGTTCGAGA | ATATTTGTTCAGAAGGTCACCATG |
| OE6A089828 | GGAGAACTGGGCTGAGCAAA | TGCCCCTGTTTCATCTTGCA |
| OE6A102071 | AGGTGGAGTGAAGAAGCCTCAC | AATTTCTCGAACAAGCCTCTGGAA |

**Supplementary Table 3** Enriched analysis of the differentially accumulated metabolites (DAMs).

|  | Compound name | VIP | P value | Q value | Log_2_(Fold change) |
| --- | --- | --- | --- | --- | --- |
| DAMs in RT vs. R group | Carbendazim | 2.60 | 0.00 | 0.01 | -4.88 |
|  | L-tyrosine | 1.55 | 0.01 | 0.13 | -2.18 |
|  | (+)-affinisine | 1.20 | 0.05 | 0.31 | -2.15 |
|  | 12(13)-EpOME | 2.36 | 0.01 | 0.10 | -1.71 |
|  | N-((-)-jasmonoyl)-S-isoleucine | 2.35 | 0.00 | 0.01 | -1.70 |
|  | 3-hydroxy-4-methoxycinnamic acid; isoferulic acid | 2.24 | 0.00 | 0.01 | -1.43 |
|  | Inositol | 1.81 | 0.01 | 0.13 | -1.33 |
|  | DL-tyrosine | 1.62 | 0.01 | 0.12 | -1.30 |
|  | Arachidonic acid | 1.92 | 0.01 | 0.16 | -1.28 |
|  | Oleocanthal | 1.72 | 0.03 | 0.27 | -1.18 |
|  | 11b-hydroxyandrost-4-ene-3,17-dione | 2.12 | 0.01 | 0.11 | -1.13 |
|  | Epifriedelanol | 1.65 | 0.01 | 0.10 | -1.09 |
|  | L-carnitine | 2.05 | 0.00 | 0.09 | -1.04 |
|  | L-histidine | 2.60 | 0.00 | 0.00 | -0.89 |
|  | Guanidineacetic acid | 1.98 | 0.00 | 0.09 | -0.88 |
|  | Ethyl gallate | 1.77 | 0.02 | 0.22 | -0.88 |
|  | Astaxanthin | 1.80 | 0.03 | 0.23 | -0.79 |
|  | Stigmasterol | 1.86 | 0.01 | 0.16 | -0.75 |
|  | L-asparagine | 1.42 | 0.04 | 0.31 | -0.75 |
|  | 5-oxoproline | 2.10 | 0.00 | 0.05 | -0.74 |
|  | 4-methylumbelliferone | 1.91 | 0.00 | 0.07 | -0.68 |
|  | Tropine acetate; 3-acetoxytropane | 1.87 | 0.01 | 0.15 | -0.63 |
|  | 4-aminobutyric acid | 1.55 | 0.03 | 0.24 | -0.58 |
|  | 5-methyl-2-furaldehyde | 2.36 | 0.00 | 0.01 | -0.53 |
|  | Tryptophan; L-tryptophan; D-tryptophan | 1.79 | 0.02 | 0.22 | -0.46 |
|  | Deethylatrazine | 2.32 | 0.00 | 0.02 | -0.42 |
|  | 3-methy-L-histidine | 1.71 | 0.03 | 0.25 | -0.40 |
|  | L-ornithine | 2.04 | 0.01 | 0.11 | -0.35 |
|  | L-arginine | 1.64 | 0.04 | 0.28 | -0.20 |
|  | Benzoic acid | 2.15 | 0.00 | 0.08 | 0.46 |
|  | 4-pyridoxolactone | 2.02 | 0.02 | 0.20 | 0.55 |
|  | Allicin | 1.51 | 0.04 | 0.30 | 0.63 |
|  | N6-isopentenyladenosine | 1.78 | 0.01 | 0.15 | 0.68 |
|  | (10E,12Z)-(9S)-9-hydroperoxyoctadeca-10,12-dienoic acid | 2.05 | 0.01 | 0.10 | 0.73 |
|  | Methyl jasmonate | 1.72 | 0.03 | 0.25 | 0.75 |
|  | Psoralidin | 1.70 | 0.02 | 0.18 | 0.76 |
|  | Melatonin | 1.74 | 0.05 | 0.32 | 0.82 |
|  | Uplandicine | 1.57 | 0.03 | 0.25 | 0.84 |
|  | Palmitoylethanolamide | 2.14 | 0.00 | 0.03 | 0.88 |
|  | Sphingosine | 1.80 | 0.02 | 0.21 | 0.89 |
|  | 3,4-dihydrocoumarin | 1.98 | 0.00 | 0.06 | 0.91 |
|  | 2-isopropyl-3-oxosuccinate | 1.10 | 0.04 | 0.28 | 0.92 |
|  | beta-nicotinamide mononucleotide | 2.08 | 0.04 | 0.30 | 0.95 |
|  | Alexine; australine | 1.79 | 0.01 | 0.16 | 1.02 |
|  | Tabernanthine | 1.76 | 0.01 | 0.16 | 1.02 |
|  | 2-picolinic acid | 2.15 | 0.00 | 0.06 | 1.09 |
|  | Adenosine 3'-monophosphate | 2.29 | 0.00 | 0.03 | 1.11 |
|  | Narciclasine | 2.55 | 0.00 | 0.00 | 1.12 |
|  | Betulin | 2.01 | 0.04 | 0.29 | 1.15 |
|  | 5-methylcytosine | 1.53 | 0.05 | 0.31 | 1.23 |
|  | Lecanoric acid | 1.16 | 0.02 | 0.22 | 1.23 |
|  | N1-methyl-2-pyridone-5-carboxamide | 1.36 | 0.02 | 0.18 | 1.27 |
|  | Deguelin | 2.16 | 0.00 | 0.07 | 1.30 |
|  | Cytosine | 1.94 | 0.00 | 0.03 | 1.33 |
|  | Serotonin | 1.30 | 0.01 | 0.12 | 1.42 |
|  | Nicotinamide | 1.98 | 0.04 | 0.28 | 1.45 |
|  | N-hydroxy tryptamine | 2.17 | 0.00 | 0.06 | 1.47 |
|  | Glycitein | 1.59 | 0.00 | 0.04 | 1.52 |
|  | D-alanyl-D-alanine | 1.92 | 0.01 | 0.13 | 1.52 |
|  | Riboflavine | 2.13 | 0.01 | 0.10 | 1.54 |
|  | Dihydrozeatin | 2.12 | 0.00 | 0.09 | 1.58 |
|  | alpha-cyperone | 2.23 | 0.02 | 0.20 | 1.99 |
|  | Loganin | 1.88 | 0.03 | 0.26 | 2.26 |
|  | Deoxyguanosine | 2.48 | 0.00 | 0.03 | 2.27 |
|  | Esculin | 2.57 | 0.00 | 0.00 | 2.45 |
|  | Hygromycin B | 1.78 | 0.01 | 0.13 | 2.50 |
| DAMs in ST vs. S group | Grandifloric acid; steviol | 2.30 | 0.00 | 0.06 | -6.01 |
|  | Carbendazim | 1.87 | 0.01 | 0.09 | -4.50 |
|  | Tetrahydroaldosterone-3-glucuronide | 2.03 | 0.00 | 0.03 | -4.04 |
|  | Savinin | 1.28 | 0.03 | 0.15 | -3.67 |
|  | Sesamolin | 2.27 | 0.00 | 0.04 | -3.25 |
|  | Maltotetraose | 2.30 | 0.00 | 0.04 | -3.13 |
|  | Stachyose | 2.30 | 0.00 | 0.04 | -3.13 |
|  | Phenylacetic acid; 4-methylbenzoic acid; M-toluic acid | 1.60 | 0.00 | 0.05 | -2.87 |
|  | Kaempferol 3-O-beta-sophoroside; cyanin | 1.66 | 0.01 | 0.10 | -2.74 |
|  | Fisetin | 2.41 | 0.00 | 0.00 | -2.39 |
|  | Myricetin | 1.62 | 0.04 | 0.18 | -2.05 |
|  | N,N'-diacetylchitobiose | 1.10 | 0.03 | 0.15 | -1.94 |
|  | Arecaidine | 1.33 | 0.03 | 0.15 | -1.92 |
|  | Genistin | 1.85 | 0.01 | 0.10 | -1.87 |
|  | 1-kestose | 1.36 | 0.00 | 0.04 | -1.85 |
|  | Canadine | 1.42 | 0.03 | 0.15 | -1.82 |
|  | Indole-3-carboxaldehyde | 2.37 | 0.00 | 0.00 | -1.64 |
|  | Styrene oxide | 2.13 | 0.00 | 0.00 | -1.60 |
|  | N-((-)-jasmonoyl)-S-isoleucine | 2.07 | 0.01 | 0.10 | -1.60 |
|  | (-)-epicatechin gallate | 1.60 | 0.02 | 0.13 | -1.57 |
|  | N,N-dimethylaniline | 1.74 | 0.02 | 0.11 | -1.48 |
|  | Serotonin | 1.27 | 0.00 | 0.04 | -1.47 |
|  | Demethoxycapillarisin | 1.77 | 0.04 | 0.16 | -1.46 |
|  | Atranorin | 1.52 | 0.05 | 0.19 | -1.40 |
|  | L-carnitine | 1.79 | 0.01 | 0.08 | -1.38 |
|  | 9-methoxy-alpha-lapachone | 1.85 | 0.01 | 0.09 | -1.37 |
|  | Methyl (indol-3-yl) acetate; methyl 3-indolylacetate | 1.69 | 0.04 | 0.17 | -1.36 |
|  | Isochlorogenic acid B | 1.60 | 0.01 | 0.10 | -1.36 |
|  | N,N-dihydroxy-L-phenylalanine | 1.69 | 0.02 | 0.12 | -1.35 |
|  | Nicotinate ribonucleoside | 2.12 | 0.00 | 0.04 | -1.35 |
|  | Perakine | 1.63 | 0.04 | 0.18 | -1.33 |
|  | 1-methyl-2-nonylquinolin-4(1H)-one | 1.24 | 0.05 | 0.19 | -1.27 |
|  | Nicotinic acid mononucleotide | 2.01 | 0.00 | 0.04 | -1.22 |
|  | 12(13)-EpOME | 1.69 | 0.02 | 0.11 | -1.21 |
|  | 3-hydroxy-4-methoxycinnamic acid; isoferulic acid | 1.77 | 0.01 | 0.08 | -1.20 |
|  | 4-methyl-5-thiazoleethanol | 1.01 | 0.04 | 0.16 | -1.18 |
|  | Schaftoside | 1.40 | 0.02 | 0.10 | -1.14 |
|  | beta-asarone | 1.61 | 0.02 | 0.10 | -1.10 |
|  | Arachidonic acid | 1.67 | 0.01 | 0.09 | -1.10 |
|  | Calystegine B2 | 1.72 | 0.01 | 0.07 | -1.07 |
|  | Allysine (6-Oxo DL-norleucine) | 1.91 | 0.01 | 0.07 | -1.06 |
|  | 4-methylumbelliferone | 1.64 | 0.01 | 0.09 | -0.98 |
|  | Isogentisin | 1.72 | 0.02 | 0.13 | -0.97 |
|  | Octyl gallate | 1.70 | 0.02 | 0.11 | -0.93 |
|  | Betaine | 1.13 | 0.04 | 0.16 | -0.90 |
|  | Trans-4-hydroxy-L-proline; 4-hydroxyproline | 1.95 | 0.01 | 0.10 | -0.88 |
|  | Alexine; australine | 1.58 | 0.05 | 0.19 | -0.87 |
|  | 5-methyl-2-furaldehyde | 2.09 | 0.00 | 0.01 | -0.86 |
|  | Serpentine | 1.66 | 0.01 | 0.10 | -0.83 |
|  | Glucofrangulin B | 1.52 | 0.04 | 0.17 | -0.83 |
|  | Ethyl gallate | 1.51 | 0.02 | 0.11 | -0.82 |
|  | Guaiacol | 1.68 | 0.01 | 0.09 | -0.80 |
|  | Oleocanthal | 1.49 | 0.05 | 0.19 | -0.76 |
|  | Epitulipinolide | 1.62 | 0.01 | 0.10 | -0.73 |
|  | 7-(4-Hydroxyphenyl)-1-phenyl-4-hepten-3-one | 1.67 | 0.01 | 0.10 | -0.68 |
|  | Scopolin | 1.59 | 0.02 | 0.11 | -0.67 |
|  | Sucrose | 1.49 | 0.03 | 0.14 | -0.66 |
|  | Palmitic acid | 1.76 | 0.01 | 0.10 | -0.62 |
|  | (-)-cinchonidine | 1.73 | 0.01 | 0.08 | -0.50 |
|  | Denudatine | 1.71 | 0.01 | 0.09 | -0.48 |
|  | Phosphoric acid | 1.81 | 0.01 | 0.08 | -0.29 |
|  | D-xylulose | 1.63 | 0.02 | 0.11 | 0.32 |
|  | p-toluenesulfonic acid | 1.56 | 0.02 | 0.12 | 0.37 |
|  | Tryptophan; L-tryptophan; D-tryptophan | 2.07 | 0.00 | 0.02 | 0.37 |
|  | Ethyl isovalerate | 1.99 | 0.00 | 0.04 | 0.44 |
|  | 5-aminovaleric acid | 1.70 | 0.02 | 0.11 | 0.53 |
|  | Levamisole | 1.64 | 0.03 | 0.15 | 0.55 |
|  | Aminomalonic acid | 1.52 | 0.02 | 0.11 | 0.55 |
|  | L-phenylalanine; D-(+)-Phenylalanine; DL-phenylalanine | 1.23 | 0.03 | 0.14 | 0.64 |
|  | 4-pyridoxolactone | 1.88 | 0.00 | 0.05 | 0.73 |
|  | Lumichrome | 1.73 | 0.04 | 0.18 | 0.79 |
|  | Vasicine | 1.65 | 0.02 | 0.11 | 0.81 |
|  | L-homoglutamic acid | 1.51 | 0.05 | 0.19 | 0.84 |
|  | Adenosine 3'-monophosphate | 2.20 | 0.00 | 0.04 | 0.88 |
|  | NG,NG-dimethylarginine dihydrochloride | 1.69 | 0.00 | 0.05 | 0.94 |
|  | Phenylacetylglycine | 1.54 | 0.03 | 0.14 | 1.05 |
|  | Pyridoxamine 5'-phosphate | 1.61 | 0.02 | 0.11 | 1.09 |
|  | Nicotinamide | 2.09 | 0.01 | 0.08 | 1.11 |
|  | L-histidine | 2.32 | 0.00 | 0.00 | 1.17 |
|  | 3-methy-L-histidine | 2.03 | 0.00 | 0.03 | 1.20 |
|  | Metanephrine | 1.54 | 0.04 | 0.18 | 1.32 |
|  | 2,3-dihydro-2-phenyl-4H-benzopyran-4-one | 1.70 | 0.01 | 0.09 | 1.45 |
|  | Tabernanthine | 1.87 | 0.01 | 0.07 | 1.52 |
|  | Esculin | 2.02 | 0.00 | 0.03 | 1.63 |
|  | N-feruloyl putrescine | 2.00 | 0.00 | 0.04 | 1.63 |
|  | Lecanoric acid | 1.38 | 0.00 | 0.04 | 1.71 |
|  | Adenosine 5'-monophosphate | 1.67 | 0.04 | 0.17 | 1.74 |
|  | DL-alanine; L-alanine | 2.02 | 0.02 | 0.11 | 1.79 |
|  | Dihydrozeatin | 2.09 | 0.00 | 0.05 | 1.92 |
|  | D-alanyl-D-alanine | 2.10 | 0.00 | 0.04 | 2.09 |
|  | S-adenosylmethionine | 1.09 | 0.02 | 0.11 | 2.15 |
|  | Narciclasine | 2.30 | 0.00 | 0.04 | 2.26 |
|  | alpha-cyperone | 2.23 | 0.01 | 0.09 | 2.58 |
|  | DL-tyrosine | 1.58 | 0.00 | 0.04 | 3.17 |
|  | 20-OH-leukotriene B4 | 2.10 | 0.00 | 0.05 | 4.14 |
|  | L-tyrosine | 1.89 | 0.01 | 0.06 | 5.35 |

S, highly sensitive germplasm; R, highly resistant germplasm; ST, highly sensitive germplasm after treatment; RT, highly resistant germplasm after treatment; VIP, variable importance in the projection.

**Supplementary Table 4** Statistics of RNA-sequencing reads mapping to olive reference genome.

| Sample replicates | Raw reads (M) | Clean reads (M) | Clean reads Q20 (%) | Clean reads Q30 (%) | Mapping rate (%) |
| --- | --- | --- | --- | --- | --- |
| S_1 | 47.51 | 47.17 | 97.75 | 93.40 | 90.35 |
| S_2 | 47.16 | 46.80 | 97.68 | 93.24 | 90.23 |
| S_3 | 42.67 | 42.36 | 97.83 | 93.51 | 89.56 |
| ST_1 | 44.60 | 44.32 | 98.08 | 94.11 | 92.05 |
| ST_2 | 47.97 | 47.63 | 97.85 | 93.63 | 91.21 |
| ST_3 | 47.30 | 46.96 | 97.69 | 93.24 | 90.01 |
| R_1 | 43.25 | 42.77 | 98.02 | 94.27 | 92.20 |
| R_2 | 43.29 | 42.90 | 97.76 | 93.32 | 92.42 |
| R_3 | 45.27 | 44.82 | 97.75 | 93.40 | 91.80 |
| RT_1 | 47.67 | 47.26 | 97.82 | 93.55 | 91.25 |
| RT_2 | 48.84 | 48.38 | 97.67 | 93.19 | 91.22 |
| RT_3 | 45.02 | 44.53 | 97.69 | 93.27 | 91.18 |

S, highly sensitive germplasm; R, highly resistant germplasm; ST, highly sensitive germplasm after treatment; RT, highly resistant germplasm after treatment.

**Supplementary Table 5** Correlation analysis of upregulated DAMs and DEGs in RT vs. R group.

| Pathway ID | Pathway name (hits number) | Hits KEGG ID of DAMs and DEGs |  |
| --- | --- | --- | --- |
| oeu04075 | Plant hormone signal transduction (254) | C02029\|C18699\|K13416\|K13422\|K13463\|K13464\|K13946\|K14431\|K14432\|K14484\|K14485\|K14487\|K14488\|K14493\|K14496\|K14497\|K14506\|K14514\|K14516 | |
| oeu00940 | Phenylpropanoid biosynthesis (130) | C00082\|K00083\|K00430\|K01904\|K09755\|K13065\|K22395 | |
| oeu04141 | Protein processing in endoplasmic reticulum (101) | K03094\|K03347\|K06689\|K09503\|K10666\|K13993 | |
| oeu04626 | Plant-pathogen interaction (82) | K05391\|K13412\|K13416\|K13433\|K13434\|K13457\|K20536 | |
| oeu04016 | MAPK signaling pathway (79) | K13416\|K13422\|K14496\|K14497\|K14514\|K14516\|K20536\|K20547\|K20604 | |
| oeu00270 | Cysteine and methionine metabolism (54) | K00640\|K00815\|K00826\|K00899\|K01611\|K01738\|K01739\|K01761\|K05933 | |
| oeu04120 | Ubiquitin mediated proteolysis (54) | K03094\|K03347\|K03869\|K06689\|K08770\|K10144 | |

DAMs, differentially accumulated metabolites; DEGs, differentially expressed genes; R, highly resistant germplasm; RT, highly resistant germplasm after treatment.
